# Investigation of the Bonding Performance and Microstructure of MOC Binders for SiO_2_ as Rock-like Composites

**DOI:** 10.3390/ma17164083

**Published:** 2024-08-17

**Authors:** Jie Jing, Hongbo Li, Xin Zheng, Kai Zhao

**Affiliations:** 1 Chongqing Institute of Green and Intelligent Technology, Chinese Academy of Sciences, Chongqing 400714, China; jingjie96@sina.cn (J.J.); zhengxin@cigit.ac.cn (X.Z.); zhaokai@cigit.ac.cn (K.Z.); 2 School of Materials Science and Engineering, Chongqing Jiaotong University, Chongqing 400074, China; 3 Chongqing School, University of Chinese Academy of Sciences, Chongqing 400714, China

**Keywords:** rock-like materials, magnesium chloride cement, microstructure, compressive strength, stiffness

## Abstract

The heterogeneity of natural rocks complicates the study of carbon sequestration within these materials and raises concerns about the reproducibility of experimental results. Consequently, identifying appropriate rock-like materials has become critical. This research examined the impact of various factors—humidity, binder content, curing period, and cold pressure—on the bond strength of magnesium oxychloride cement (MOC) through orthogonal testing. The tests utilized a molar ratio of MgO to MgCl_2_-6H_2_O to H_2_O of 7:1:18. Both X-ray diffraction (XRD) and scanning electron microscopy (SEM) were employed to analyze the hydration reactions of MOC and to explore the correlation between the microstructure of the SiO_2_-MgO-MgCl_2_ system and its bonding characteristics. The findings indicated that a 5% relative humidity enhances the 7-day bond strength of MOC. Moreover, increasing the curing humidity to 60% relative humidity supports the ongoing hydration of the strength-contributing phases. A binder content ranging from 15% to 25% proved optimal, yielding samples with superior strength and stiffness. While cold pressing initially enhances the bonding properties of MOC, solution loss during the process adversely affects its long-term bonding characteristics. From a mechanical standpoint, the silica-magnesium oxide-magnesium chloride system demonstrates exceptional early strength and resilience, positioning it as a promising rock-like material system.

## 1. Introduction

In rock mechanics, testing natural samples requires substantial manpower and material resources, from sampling and transportation to molding, complicating sample preparation. Moreover, these samples generally only permit a single use, precluding repeated testing post-destruction. Typically, rock mechanics tests involve multiple experimental groups and variables to control specific factors, necessitating a high degree of similarity among some samples [1]. Nevertheless, the inherent complexity and diversity of natural rock compositions and structures pose challenges. Traditional core drilling methods significantly disturb the samples, obscuring their internal structures and resulting in varied mechanical properties [2]. Even when samples are extracted from the same location under identical conditions, the resultant data dispersion remains substantial. Although this dispersion may occasionally meet experimental requirements, the inherent differences in the samples introduce unavoidable errors [3]. Consequently, the investigation of rock-like materials to simulate natural rocks has emerged as a prominent research area in recent years.

Three primary requirements exist for simulating natural rock materials: (1) mechanical properties akin to natural rocks, (2) similar composition, and (3) comparable microstructure. Currently, four types of raw materials are predominantly used in rock mechanics research: photosensitive resin, plastic, powder-based, and cement (gypsum) materials. Among these, powder and resin materials are the most commonly utilized rock-like materials [4,5,6,7,8,9,10,11]. Numerous scholars [12,13,14,15,16,17] have employed epoxy resin as an aggregate in producing transparent, pre-fabricated, cracked, rock-like samples. The transparency of resin is beneficial for studying crack propagation characteristics. However, epoxy resin, when used as an aggregate, exhibits significant brittleness, necessitating additional hardeners and presenting notable discrepancies in composition compared to natural rocks. While resin materials offer visibility, their microstructural fidelity to natural rocks is limited, primarily simulating only the brittleness of natural rocks without capturing detailed microstructural features of crystal intergranular formations. Conversely, models made from powder materials, although not permitting direct observation of internal crack development, more closely resemble the fine microstructure of natural rocks. Unlike photosensitive resins, powdered materials like gypsum and sand closely mimic the natural rock powders in terms of particle size and surface roughness, thanks to adhesive bonding printing.

**Figure 1:**
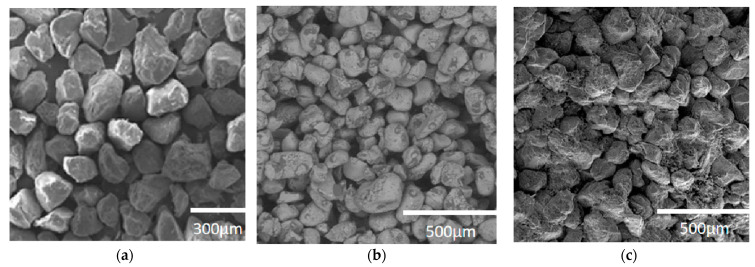
SEM images of sand-type 3D-printed rock-like models and natural sandstone: (**a**) GS19-type sand; (**b**) silica sand; (**c**) Berea sand.

Sand materials, such as silica sand, pearl sand, and quartz sand, are a subset of powder materials that can more closely simulate the microstructure of natural rocks, particularly natural sandstone. This is evident in the crystal particle characteristics of sand-based materials which closely mimic those of natural formations. Figure 1 displays SEM images of a sand-type 3D-printed rock-like model alongside natural sandstone [18,19], with Figure 1a,b depicting the 3D-printed model and Figure 1c showcasing natural sandstone. These images reveal that the particle arrangement in the 3D-printed model is sparser than in natural sandstone. Additionally, the particles in the 3D-printed model are not notably cemented, contributing to its lower density and cement content. These factors are primarily responsible for the reduced strength observed in numerous studies of sand-type 3D-printed models.

Binders for rock-like materials fall into two primary categories: organic and inorganic. Among organic binders, epoxy resin stands out for its effective cementing properties, particularly in repairing and reinforcing fissured rocks [20,21,22], although its composition vastly differs from natural rock binders [23]. In contrast, the most notable inorganic binders include various mineral salts, such as alumino-silicates and silicates [24], as well as sodium silicate [25,26]. However, these binders generally require high temperatures (above 600 °C) to achieve sufficient silicate cementation, which can alter the physicochemical properties of the rock-like materials. This analysis indicates that current binder systems often face challenges related to composition dissimilarity or demanding manufacturing conditions in simulations of natural rocks.

Magnesium oxychloride cement (MOC), also referred to as sorrel cement or magnesia cement, is a ternary composite system consisting of MgO, MgCl_2_, and H_2_O [27]. This system undergoes hydration reactions that produce crystalline phases, with the most significant being Phase 3 (3Mg(OH)_2_·MgCl_2_·8H_2_O), Phase 5 (5Mg(OH)_2_·MgCl_2_·8H_2_O), and Mg(OH)_2_. Among these, Phase 5 is notable for providing superior mechanical properties [28]. Recent studies summarized in Table 1 demonstrate that the mechanical properties and stability of Phase 5 are primarily determined by the MgO/MgCl_2_ molar ratio. Optimal product strength and mechanical properties are achieved when this ratio is 7, and the MgCl_2_ concentration is 2.25 mol·kg^−1^, corresponding to a ternary molar ratio of 7:1:18.

This study uses magnesium oxychloride (MOC) as the binder and silica as the primary material to investigate their viability as rock-like materials. Using MOC eliminates the need for high-temperature processing, and its hydration products can react with natural rock components, closely mimicking the properties of natural rock. The research evaluates the impact of various humidity maintenance methods and binder concentrations on the compressive strength of the samples from both microscale and macroscale perspectives, aiming to provide a scientific and reliable theoretical foundation for the application of this material system in rock-like applications.

## 2. Materials and Methods

**Figure 2:**
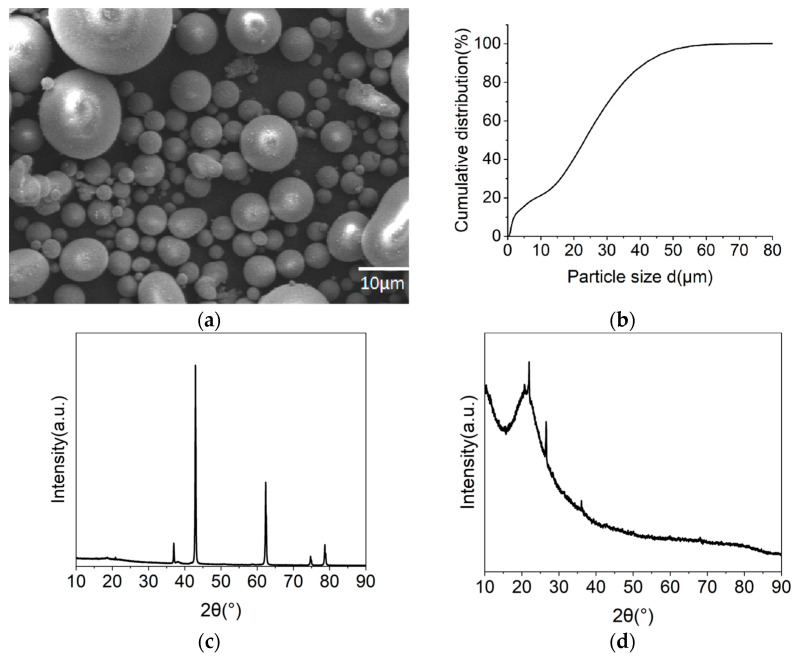
SEM (**a**) and particle size distribution of raw material SiO_2_ (**b**); XRD results of MgO (**c**) and SiO_2_ (**d**).

In this study, HN-Mg50 type MgO powder with a scorch loss of 0.4% was utilized. Spherical SiO_2_, possessing a median particle size of 24 μm, was examined; the SEM images and particle size test results are displayed in Figure 2a,b. The MgCl_2_·6H_2_O used was analytically pure and had been stored in sealed conditions for an extended period. The composition of the raw materials and the XRD test results are presented in Table 2 and Figure 2c,d.

The raw materials were prepared according to Table 3, and the powder was canned and mixed on a GMS3-4 drum ball mill for 1 h. The mixed powder was poured into the mixer, mixed with magnesium chloride solution, and stirred for 15 min. The stirred slurry was poured into a steel mold with a diameter of 20 mm and vibrated on a D300 sifter for 30 min. The surface of the mold was sealed and placed for 8 h for demolding, and then placed in a sealed bag for 24 h to obtain the sample. S10, S20, and S30 samples were set up with 3 sets of cold pressing process during the sample making process, and the duration of cold pressing was 1 h (Table 4). The samples were placed in a YH-40B constant temperature curing box, set the temperature at 25 °C and the humidity at 5%, 60% and 90%, and were cured until the 7, 14 and 28 days.

Testing methods:

(1) Mechanical properties test

Use TM5105 universal testing machine in accordance with the speed of 1 mm/min for uniaxial compressive testing of samples under different maintenance environment.

(2) Microstructure characterization

Quantitative analysis of the internal binder composition by XRD, SEM-EDS, and other test results to study the effect of humidity and binder content on the type and content of hydration products.

(3) Density test

The mass and volume of the samples maintained for 7 days were measured, and their densities were calculated by the mass method to study the effect of different humidity on the density of the samples.

Before conducting the mechanical property tests and density measurements, the samples were machined into standard uniaxial compressive cylindrical test pieces with a height-to-diameter ratio of at least 2:1.

## 3. Results

**Figure 3:**
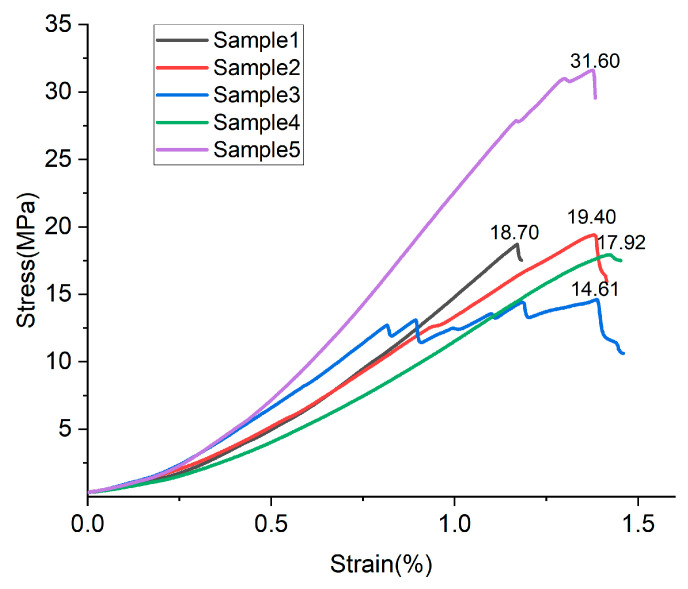
Stress–strain curves of S20-M samples on day 7.

To assess the measurement uncertainty of the study, the compressive test data from the samples were statistically analyzed to exclude those with apparent defects or outliers, after which an average was computed for the remaining data. Taking the five measurements of the S20-M samples on day 7 as an example (Table 5), it was found in the stress–strain curves (Figure 3) that the four samples, except for Sample 3, conformed to the general pattern of uniaxial compression. The stress–strain curve of Sample 3 shows a wavy shape before the peak, which indicates that there were obvious defects inside the sample and that it should be rejected. The data of Sample 5 are obviously large compared with Samples 1, 2, and 4, and is an outlier and should be rejected. Both of the above rejected values are caused by the uneven distribution of the binder during the sample-making process. The average of the three retained measurements yields a compressive strength of 18.67 MPa for the S20-M sample on day 7.

### 3.1. Effect of Humidity on Bonding Performance

**Figure 4:**
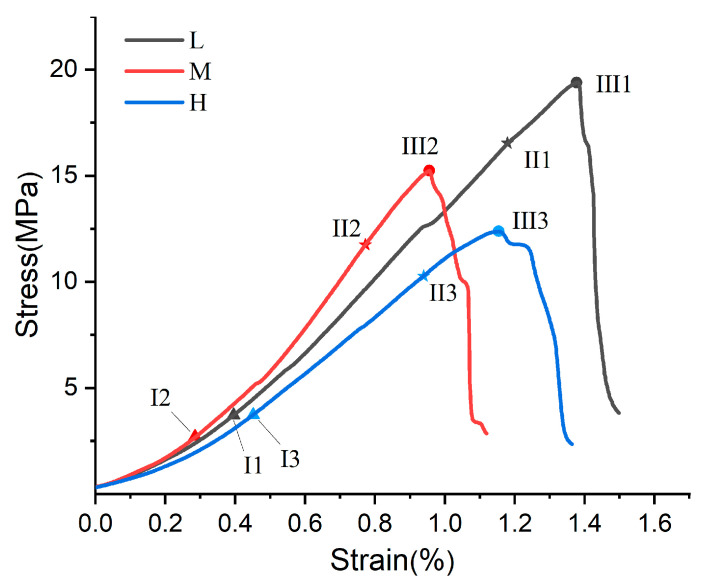
Compression curves of S20 for 7 days at 3 conservation humidity levels.

Figure 4 illustrates the relationship between sample strength and humidity, denoted as L > M > H, indicating that the MOC bond strength decreases with increasing humidity. The depressed compression segments (0-I1, 0-I2, 0-I3) evident in the curves could be due to the complex interaction between the sample and the tester head [35], or the closure of microcracks under compression within the sample. To mitigate the influence of the former, the samples were standardized prior to conducting the compression tests. In this study, the depressed compression section in the initial part of the curve is mainly attributed to the effect of compression closure of cracks inside the sample. Notably, the 0-I2 segment is the shortest, suggesting that a relative humidity (RH) of 60% enhances the densification of the internal structure of the sample. The slope of the stress–strain curves during the elastic phase follows the order I2-II2 > I1-II1 > I3-II3, which demonstrates that the stiffness of the samples is greatest at 60% conservation humidity. Beyond the yield point, the curves display convex plastic segments (II1-III1, II2-III2, II3-III3) before reaching peak strength. The sample conditioned at 5% RH exhibited the highest ultimate strength. Overall, the analysis of the stress–strain curves indicates that 60% RH increases the stiffness, while 5% RH enhances the strength of the samples.

**Figure 5:**
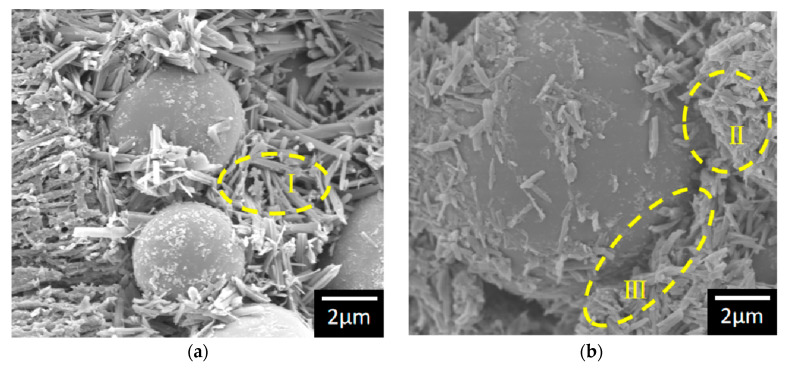
SEM images of S20 at different conservation humidity: (**a**) L; (**b**) M (note: the water content of the sample in the H conservation environment is too high to be observed under the SEM).

The S20 samples under different conservation humidity were analyzed using scanning electron microscopy (SEM) to investigate the impact of humidity on the microstructure of MOC, with the findings presented in Figure 5. This figure reveals that the surface or the end of the needle-rod bar crystals showed loose blocky and lamellar structure at I and II. Research cited in reference [36] indicates that conditioning temperatures below 30 °C promote the formation of flaky and layered Mg(OH)_2_, which, considering MOC’s limited water resistance, leads to the transformation of some of Phase 3 and Phase 5 into Mg(OH)_2_ over a period of up to 7 days. This transformation underlines the instability of Phase 5 crystals, as hypothesized. Furthermore, a comparison of Phase 3 and Phase 5 decomposition at I and II demonstrates that higher humidity accelerates the conversion to Mg(OH)_2_, resulting in increased formation of this compound.

As observed in Section III of Figure 5b, the increase in the conversion of Phase 3 and Phase 5, which contribute to structural strength, to Mg(OH)_2_ leads to the loosening and cracking of the binder’s encapsulation of spherical silica at the bonding interface. This phenomenon is attributable to the decomposition of a portion of Phase 5—which plays a crucial role in bonding—into structurally loose magnesium hydroxide, resulting in a loss of bonding properties. Consequently, the strength of the sample diminishes as the conditioning humidity increases.

**Figure 6:**
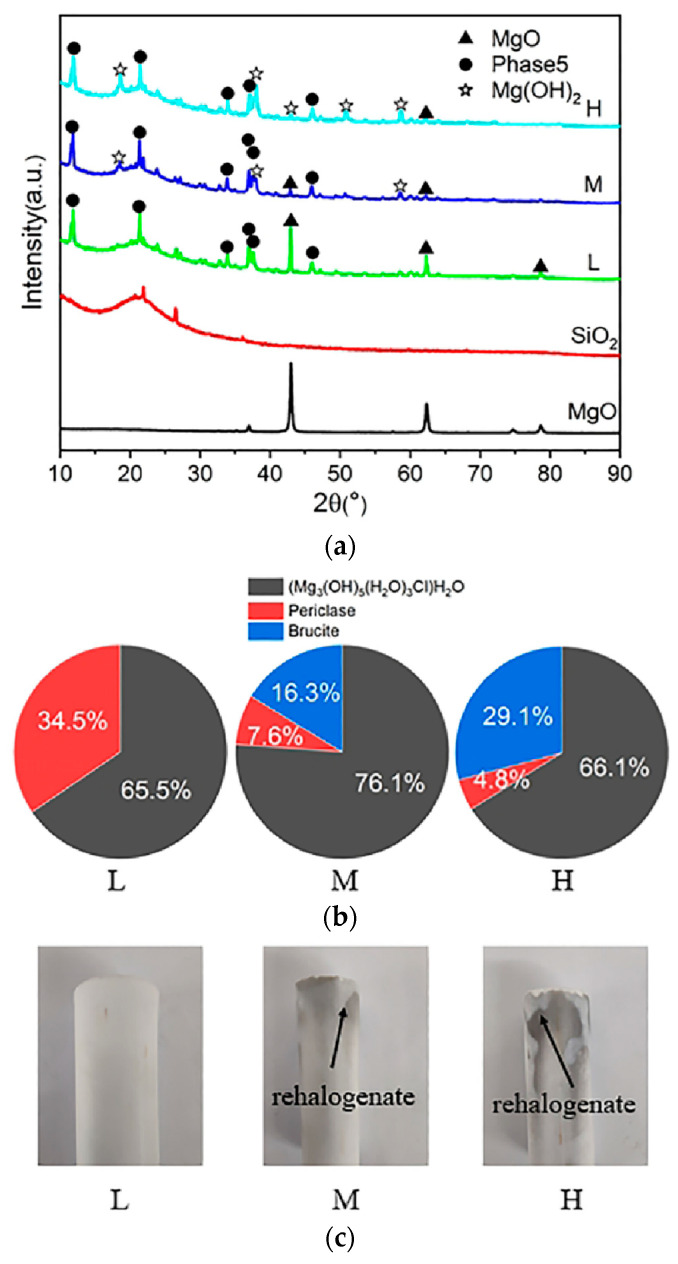
XRD plots (**a**) and semi-quantitative analysis (**b**) of S20 at three conservation humidities; (**c**) S20 rehalogenation at three humidities.

X-ray diffraction (XRD) analysis, presented in Figure 6a, reveals that as humidity increases, the intensity of the magnesium oxide peak at 2θ = 42.97 gradually decreases, transitioning into an Mg(OH)_2_ peak. Concurrently, Mg(OH)_2_ peaks at 2θ = 18.58, 38.07, 50.88, and 58.68, and becomes significantly more pronounced. Further analysis using semi-quantitative XRD data from Figure 6b indicates that the hydride content of Phase 5 in S20-M is the highest, followed by S20-H, with S20-L showing the lowest. This suggests that a moderate increase in conservation humidity favors the formation of Phase 5 compounds. However, increased humidity also leads to the formation of Mg(OH)_2_, elucidating the impact of humidity on the microstructural composition. Analysis shows that Mg(OH)_2_ primarily forms from two sources: decomposition of part of Phase 5 upon exposure to water and the reaction of residual internal magnesium oxide with water. From a compressive strength perspective, the increase in Phase 5 content due to higher maintenance humidity does not enhance the MOC bond strength. This underscores that the bonding performance of the binder system is predominantly influenced by the relative contents of Phase 5 and magnesium hydroxide, with the detrimental effects of magnesium hydroxide being significant.

The principal hydride in magnesium oxychloride cement (MOC) is identified as Phase 5 crystal, which itself does not absorb moisture. However, under the influence of water, Phase 5 decomposes into Mg(OH)_2_ and MgCl_2_. Subsequently, when the product’s surface absorbs moisture, this hydrated Phase 5 breaks down, releasing MgCl_2_ as freely soluble chloride salts (either MgCl_2_-xH_2_O or MgOHCl-xH_2_O). These salts, characterized by high water solubility and hygroscopicity, in humid environments, absorb atmospheric moisture and deliquesce, forming aqueous solutions that manifest as water beads on the product’s surface. Moreover, the salts located within the product absorb moisture through capillary action and migrate towards the surface. Consequently, in humid conditions, the product surface gradually darkens. Figure 6c demonstrates that, while samples stored at 5% relative humidity (RH) did not exhibit rehalogenation, those at 60% and 90% RH did, with the effect more pronounced at 90% RH, indicating that higher humidity levels exacerbate the decomposition of Phase 5 into Mg(OH)_2_ and MgCl_2_, thus intensifying the rehalogenation process.

In the short term, conditioning at 5% RH enhances the strength of the samples. This is primarily because low humidity effectively suppresses the formation of magnesium hydroxide, thereby mitigating its adverse impact on the bonding performance of MOC.

### 3.2. Influence of Age of Maintenance Period on Bonding Properties

**Figure 7:**
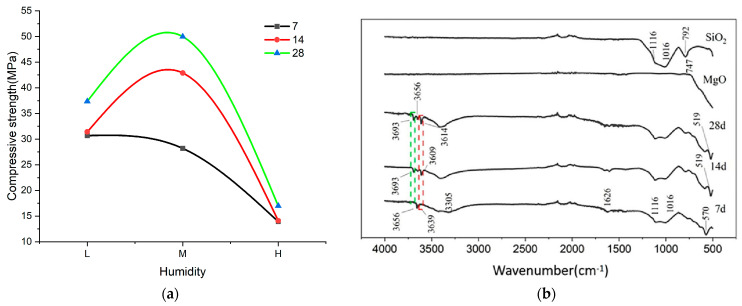
(**a**) Intensity of 7.5 MPa cold-pressed S20 at 3 humidities at 7 days, 14 days, and 28 days; (**b**) FTIR spectra of 7.5 MPa cold-pressed S20-M after 7, 14, and 28 days (green box: the O-H infrared absorption peak in Mg(OH)_2_; red box: the O-H infrared absorption peak in Phase 5).

Figure 7 depicts the compressive strength of cold-pressed S20 at 7.5 MPa after 7, 14, and 28 days across three humidity levels. Observing the graph, the strength of the samples at 7 days exhibits a decreasing trend with higher humidity. Over 14 and 28 days, the strength initially increases and then declines with rising humidity. Notably, the samples perform best at 60% RH, showing the most substantial improvement, suggesting it enhances the long-term bonding performance of MOC.

In Figure 7b, the peaks at approximately 1116 cm^−1^, 1016 cm^−1^, and 792 cm^−1^ correspond to Si-O stretching vibrations of SiO_2_. The reduced intensity of these absorption peaks suggests increased polymerization of the MOC binder over the conservation period, thereby lowering the frequency of Si-O stretching vibrations in SiO_2_ and indicating enhanced bonding strength to silica particles. Apart from the Si-O peak, the absorption spectrum at 7 days reveals prominent bands at 3656 cm^−1^, 3639 cm^−1^, 3305 cm^−1^, 1626 cm^−1^, and 570 cm^−1^. The band at 3656 cm^−1^ corresponds to free hydroxyl (-OH) stretching vibrations, while the weaker band at 3639 cm^−1^ indicates non-aqueous hydroxyl (-OH) stretching vibrations specific to Phase 5 crystals. The broad absorption at 3305 cm^−1^ is attributed to crystalline water molecule stretching vibrations in Phase 5, whereas the peak at 1626 cm^−1^ relates to their bending vibrations. Finally, the peak at 570 cm^−1^ is likely due to CO_2_ exposure during sample preparation or conditioning.

Comparison of the infrared absorption spectra at 7, 14, and 28 days reveals increasing intensity of Phase 5 peaks at 3639 cm^−1^, 3609 cm^−1^, and 3614 cm^−1^, respectively, indicating growth of Phase 5 crystals over time. The intensity of the Phase 5 peak significantly increases from 7 to 14 days, with a smaller increment from 14 to 28 days, suggesting a decrease in residual magnesium oxide and diminishing Phase 5 production as curing progresses. The displacement of the Phase 5 characteristic peak suggests varying amounts of crystallized water within the sample. At 3656 cm^−1^, the free hydroxyl (-OH) absorption peak initially decreases, then increases, reflecting ongoing hydration reactions and water absorption into the conservation environment. Initially, higher internal residual magnesium oxide at 14 days requires more water for hydration, yielding magnesium hydroxide, Phase 5, and other products, weakening the free hydroxyl absorption peak. Once a critical point is surpassed, reduced moisture demands and increased internal free water enhance the free hydroxyl absorption peak. Additionally, the O-H infrared absorption peak in Mg(OH)_2_ at 3693 cm^−1^ increases with conservation period age, indicating greater magnesium hydroxide formation.

Differences in the characteristic peaks at 570 cm^−1^ observed in the 7-day FTIR spectrum compared to those at 519 cm^−1^ in the 14-day and 28-day FTIR spectra were noted, attributed to CO_2_ exposure. The absorption peaks’ intensity at 14 and 28 days was notably stronger than at 7 days, accompanied by a shift towards lower frequencies. This variation is attributed to CO_2_ adsorption from the curing environment by the MOC binder. As the curing period progresses, cumulative CO_2_ adsorption increases, thereby enhancing the detected absorption peaks. The consistent intensity of the characteristic peaks on days 14 and 28 suggests CO_2_ adsorption may have saturated during this period.

In terms of the curing period duration, the bonding properties of MOC improve with increasing curing time. The 60% RH environment provides the necessary moisture for subsequent hydration reactions and serves as raw material for replenishing phase 5, favoring the long-term bonding performance of MOC.

### 3.3. Effect of Binder Content on Bonding Performance

**Figure 8:**
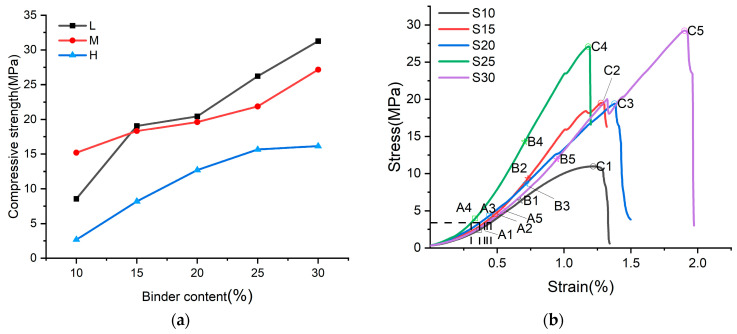
(**a**) Relationship between bond content and compressive strength of 7 days; (**b**) Stress–strain curves of samples with five binder contents at 5% RH at 7 days.

Figure 8a illustrates that the compressive strength of samples after 7 days increases with higher binder content across all three curing humidity levels. This phenomenon is attributed to Phase 5 crystals, which significantly contribute to sample strength; higher binder content results in increased Phase 5 production. Interactions between Phase 5 crystals and spherical silica encapsulation enhance the uniaxial compressive strength of the samples.

Figure 8b reveals that varying binder contents resulted in distinct mechanical properties of the samples after 7 days. During loading, all curves exhibited distinct phases: pressure-dense (0A1, 0A2, 0A3, 0A4, 0A5), elastic (A1B1, A2B2, A3B3, A4B4, A5B5), and plastic (B1C1, B2C2, B3C3, B4C4, B5C5), culminating in brittle fracture. In the pressure-tight stage, S25 corresponded to the shortest 0A4, indicating tighter internal packing of Phase 5 spherical silica and fewer internal macropores in the 25% binder content sample. The stress–strain curve slope relationship in the elastic stage shows: S25 > S15 ≈ S20 ≈ S30 > S10, indicating the highest stiffness in the 25% binder sample, the lowest in 10%, and minimal difference among other groups. This suggests stiffness initially increases then decreases with binder content. At the plastic phase end, S30 > S25 > S20 ≈ S15 > S10, indicating peak compressive values increase with binder content.

**Figure 9:**
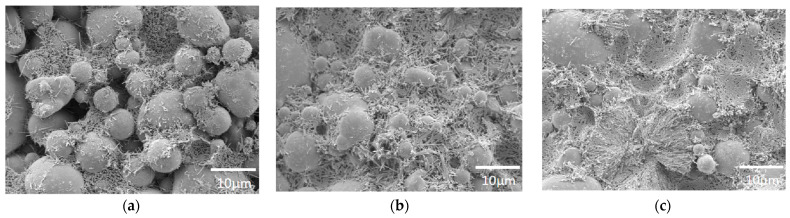

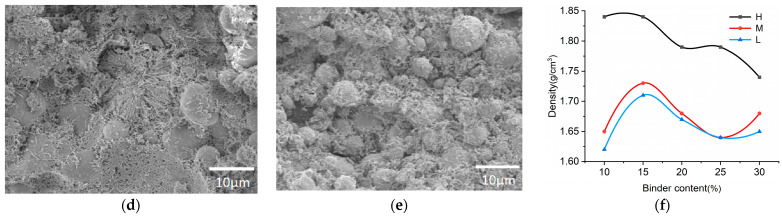
Sectional micrographs of samples with different binder contents: (**a**) S10; (**b**) S15; (**c**) S20; (**d**) S25; (**e**) S30; sample density versus binder content (**f**).

From Figure 9a–e, it is evident that increasing the binder results in denser and more compact packing of spherical silica microspheres. This observation aligns with the principle that sample strength correlates positively with binder content. However, the binder necessitates more space, thereby increasing the gaps between the silica particles. Figure 9f density curves indicate a peak in sample density within the 10–25% binder content range under conditions L and M, with maximum density achieved at 15% binder content. The density increase is attributed to the binder filling gaps, however, densities decline beyond the critical binder content for optimal gap filling. In the 25–30% binder range, density rises due to higher initial moisture content at 30% binder. Samples under humidity condition H exhibit significantly higher density profiles compared to conditions L and M, showing a decreasing trend with increasing binder content. This is because humidity condition H elevates internal water content, leading to increased formation of porous magnesium hydroxide with higher binder content, resulting in reduced density.

In terms of microstructure and mechanical properties, a binder content range of 15% to 25% appears optimal. This range facilitates improved bonding between silica particles and enhanced pore filling.

### 3.4. Effect of Cold Pressing on Bonding Properties

**Figure 10:**
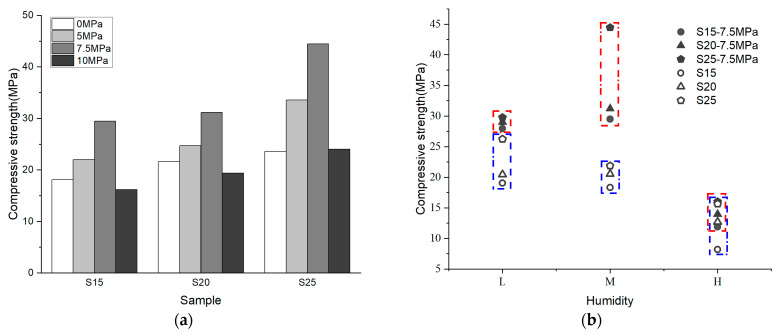
(**a**) Effect of cold compression molding process on 7-day strength of samples; (**b**) comparison of 7.5 MPa cold compression molding sample’s and vibration molding sample’s 7-day strengths (red box: dates of cold compression molding samples in the same humidity; blue box: dates of samples without cold compression molding in the same humidity).

The mechanical test results from the 7th day of cold-pressed molded samples S15, S20, and S25 were compared with those from samples made by vibration compaction (0 MPa), as depicted in Figure 10a. The compressive strength of the samples on the 7th day continued to increase with higher binder content. According to the compressive test results, samples subjected to cold compression molding at 5 MPa and 7.5 MPa exhibited improved 7-day strength. Notably, cold compression at 7.5 MPa showed the most significant enhancement, nearly doubling the strength of the S25 group samples. Conversely, samples treated with 10 MPa cold pressing showed reduced strength compared to vibration-compacted samples, suggesting excessive compression led to moisture or binder component loss within the material system, resulting in decreased strength. Although the density of the 10 MPa cold-pressed molded samples increased, their strength decreased, highlighting that the chemical reaction of MOC itself primarily influences MOC bond strength and serves as a significant factor.

Compared to the strength trend observed in vibration-molded samples with increasing humidity (Figure 10b), which showed a decline, the strength of cold compression-molded samples improved. These samples exhibited an initial increase followed by a decrease with rising humidity, with the most significant strength increase observed at 60% RH after 7 days. The enhancement can be attributed to the cold compression molding process, which enhances sample density and consequently compressive strength. Additionally, this process alters the composition of the mixture, particularly the moisture or magnesium chloride solution content, affecting the type and quantity of MOC hydration products, and thus bonding properties. Conversely, samples conditioned at 90% RH post-cold pressing showed insignificant strength improvement, indicating unsuitability for conditioning MOC-bonded samples due to their poor water resistance. At the chemical level, phase 5 primarily contributes to bond strength, but it is prone to converting to magnesium hydroxide in high-humidity conditions, leading to strength loss. Consequently, even after curing cold-pressed samples at 90% RH for 7 days, minimal to no strength increase was observed.

**Figure 11:**
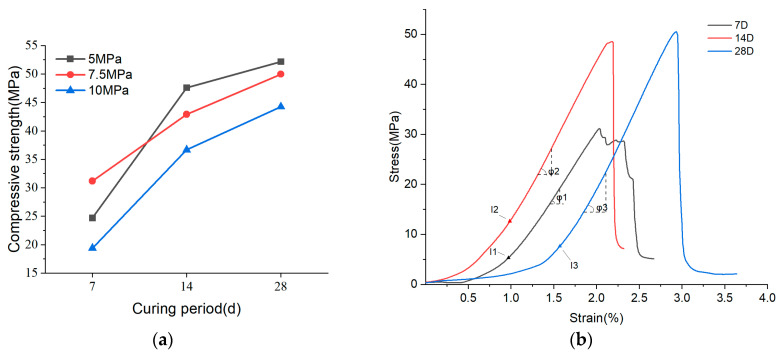
Effect of cold pressing on the long-term strength of S20-M samples (**a**); stress–strain curves of S20-M samples at 7, 14, and 28 days (**b**).

Figure 11a illustrates the rapid initial growth of MOC bond strength within the first 14 days, consistent with MOC’s strong early bonding capability. This indicates a relatively rapid hydration reaction in the MOC ternary system, generating a substantial amount of Phase 5 hydride, which enhances strength. By day 14, the strength rankings among the three samples were S20-5 MPa > S20-7.5 MPa > S20-10 MPa. The S20 sample cold-pressed at 5 MPa achieved a strength of 47.59 MPa by day 14, nearly doubling from 24.69 MPa at 7 days. By day 28, all samples showed increased strength, though the enhancement was less pronounced than in the initial 14 days, attributed to the early rapid hydration of the MOC ternary system. The strength hierarchy at 28 days remained unchanged from that at 14 days, continuing to show that higher cold-pressing strength correlated with lower compressive strength. This trend may stem from material loss during cold pressing, resulting in insufficient water and magnesium chloride for long-term curing, thus impeding Phase 5 formation. These findings suggest that excessive cold pressing hampers the development of MOC’s long-term bonding properties.

Figure 11b indicates that the densification index sequence 0I1 < 0I2 < 0I3 suggests an increase in internal pore count with prolonged curing period. This is attributed to two main factors: firstly, the rise in rod-like Phase 5 crystals, which segment the silica aggregates into smaller pores; secondly, the formation of loose layers of magnesium hydroxide as the curing period extends, further contributing to pore generation. Thus, the number of pores within the MOC-bonded samples actually increases over extended curing times. Additionally, the figure shows φ3 > φ2 > φ1, implying tan φ3 > tan φ2 > tan φ1. This signifies an increase in sample stiffness with prolonged curing, likely due to the increased presence of Phase 5 crystals. Compared to the 7-day samples, both the 14-day and 28-day samples exhibited pronounced brittle fracture after the compressive strength sharply declined towards zero. This suggests that prolonged conditioning not only enhances strength but also increases the brittleness of MOC samples.

**Figure 12:**
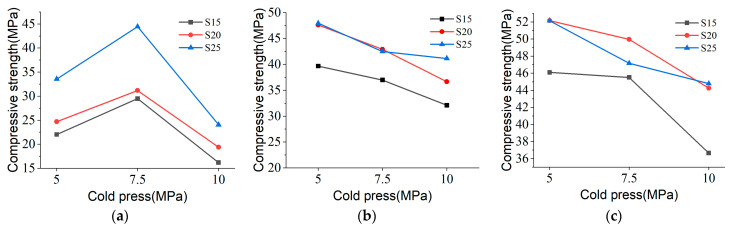
Effect of cold compression-molded samples on strength of samples cured to (**a**) 7 days, (**b**) 14 days, and (**c**) 28 days.

The compressive strength of S15, S20, and S25 at 7 days exhibited an initial increase followed by a decrease with increasing cold pressure, as depicted in Figure 12a, reaching a peak at 7.5 MPa. Figure 12b,c show that at 14 days and 28 days, respectively, compressive strength decreased with higher cold pressing, with the maximum observed at a minimal cold pressing of 5 MPa. These observations suggest that cold pressing may not be optimal for long-term bond strength development with MOC binders. This could be attributed to moisture loss from the material system during cold pressing, leading to insufficient hydration of the MOC ternary system and thereby reducing the formation of Phase 5 crystals over extended periods.

**Figure 13:**
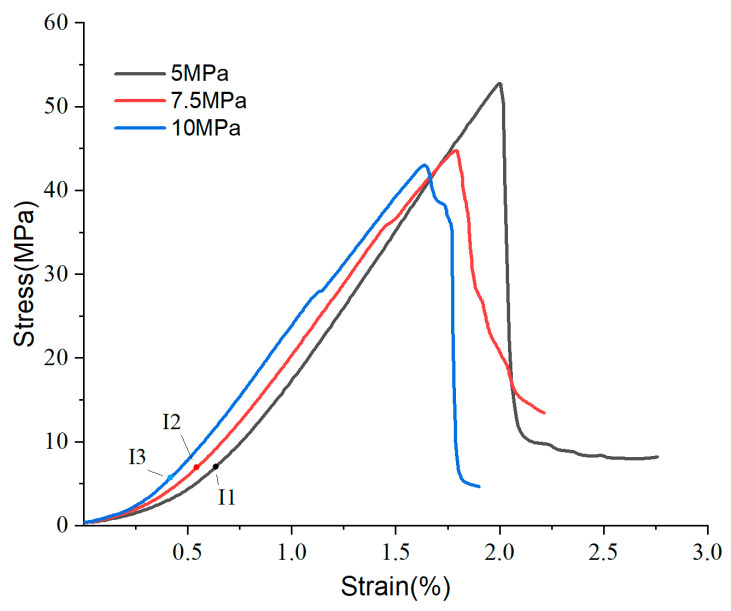
Stress–strain curves of S20-M at 28 days after three cold-pressing treatments.

From Figure 13, it is evident that the modulus of elasticity of the samples remains nearly unchanged, while brittleness decreases with increasing cold pressing. This phenomenon correlates with a reduction in the relative content of Phase 5 within the sample due to higher cold pressing pressures. Comparison of the compaction phases across each stress–strain curve reveals that 0I1 < 0I2 < 0I3, indicating that pore size decreases within the sample as cold pressure increases. This is attributed to increased contact between silica aggregate particles under higher cold pressing pressures.

From the experimental findings, suitable cold pressing enhances the short-term bond strength of MOC. However, the loss of solution and other components during the cold pressing process adversely impacts the subsequent hydration reaction, thereby hindering the long-term adhesive properties of MOC.

## 4. Conclusions

This article investigated the influence of humidity on the hydration reaction mechanism of MOC, its binder content as a rock-like material, and the feasibility of cold pressing to enhance sample density, leading to the following key findings:

(1) Humidity significantly impacts the hydration reaction of the MOC system, affecting Phase 5 content and bond strength. Specifically, MOC samples exhibited maximum 7-day bond strength at 5% RH and maximum 14- and 28-day bond strength at 60% RH;
(2) Bond strength, sample stiffness, and brittleness of MOC increase with curing age, attributable to continuous hydration and Phase 5 accumulation;
(3) Increasing binder content enhances sample strength, while sample stiffness initially increases and then decreases. Higher binder content primarily enhances bonding and gap filling, and initially increasing sample stiffness, however, excessive binder enlarges pores between aggregate particles, reducing stiffness;
(4) Cold pressing improves early bonding properties of MOC but adversely affects later bonding properties.

In summary, MOC bonded with SiO_2_ can achieve strength comparable to medium-soft rock, showing promise for high-fidelity rock simulation applications. Nonetheless, there remains a substantial density gap between this material system and natural rock, necessitating further research to optimize molding density.

## Figures and Tables

**Table 1:** Summary of some magnesium chloride cement-proportioning studies.

Authors	MgO/MgCl_2_	C_MgCl_2__ (mol·kg^−1^)	Pros and Cons
Matkovlć, et al. [29]	5	4.27	Phase 5 product theoretical ratios, incomplete reaction, residual unreactedMgO
Chau and Li. [30]	6.8	4.63	Best mechanical properties
Li, et al. [31]	7~9	-	Advantageous product strength
Li and C.K. [32]	11~17	3.68~4.63	In order to satisfy the ease of reconciliation, the concentration of the magnesium chloride solution depends to a large extent on the MgO/MgCl_2_
Ye, et al. [33]	-	<3.70	Phase 3 disappears, Phase 5 is the dominant crystal
Zhou, et al. [34]	-	1.47~2.25	Phase 5 is stable in magnesium chloride solutions from 1.47 to 2.25 mol·kg^−1^ and 2.25 is the lowest concentration at which Phase 3 occurs

**Table 2:** Chemical compositions of the raw materials used.

SiO_2_	Components	SiO_2_	Na^+^	K^+^	Ca^2+^	Mg^2+^
	Mass fraction (%)	99.9	0.005	0.0015	0.0015	0.001
MgO	Components	MgO	CaO	Cl^−^	HCl insoluble	
	Mass fraction (%)	99.2	0.03	0.02	0.02	
MgCl_2_·6H_2_O	Components	MgCl_2_·6H_2_O	SO4^2−^	PO4^3−^	Ca^2+^	Fe^3+^
	Mass fraction (%)	≥98	≤0.005	≤0.001	≤0.05	≤0.0005

**Table 3:** Material proportion and curing conditions used in this study.

Sample	Proportion		Curing Conditions	
	Si_2_O:MgO (Mass Ratio)	MgO:MgCl_2_:H_2_O (Molar Ratio)	Temperature (°C)	Relative Humidity
S10	9:1	7:1:18	25	L: 5% RH M: 60% RH H: 90% RH
S15	8.5:1.5			
S20	8:2			
S25	7.5:2.5			
S30	7:3			

**Table 4:** Cold pressing process design.

Sample	Cold Compression (MPa)	Cold Pressing Time (h)
S15	5	1
S20	7.5	
S25	10	

**Table 5:** Uniaxial compressive measurements of S20-M on day 7.

S20-M	Compressive Strength (MPa)	Retained Average (MPa)
Sample 1	18.70	18.67
Sample 2	19.40	
Sample 3	14.61	
Sample 4	17.92	
Sample 5	31.60	

## Data Availability

The original contributions presented in the study are included in the article, further inquiries can be directed to the corresponding author.
